# Prevalence of *Cryptosporidium* spp., *Enterocytozoon bieneusi*, *Encephalitozoon* spp. and *Giardia intestinalis* in Wild, Semi-Wild and Captive Orangutans (*Pongo abelii* and *Pongo pygmaeus*) on Sumatra and Borneo, Indonesia

**DOI:** 10.1371/journal.pone.0152771

**Published:** 2016-03-31

**Authors:** Anna Mynářová, Ivona Foitová, Martin Kváč, Dana Květoňová, Michael Rost, Helen Morrogh-Bernard, Wisnu Nurcahyo, Cathleen Nguyen, Supriyadi Supriyadi, Bohumil Sak

**Affiliations:** 1 Faculty of Science, University of South Bohemia in České Budějovice, České Budějovice, Czech Republic; 2 Institute of Parasitology, Biology Centre, Czech Academy of Sciences, České Budějovice, Czech Republic; 3 Department of Botany and Zoology, Faculty of Science, Masaryk University, Brno, Czech Republic; 4 Faculty of Agriculture, University of South Bohemia in České Budějovice, České Budějovice, Czech Republic; 5 Faculty of Economics, University of South Bohemia in České Budějovice, České Budějovice, Czech Republic; 6 Outrop, The Orangutan Tropical Peatland Project, Sabangau, Indonesia; 7 Department of Parasitology, Faculty of Veterinary Medicine, Gadjah Mada University, Yogyakarta, Indonesia; 8 UMI - Saving of Pongidae Foundation, Brno, Czech Republic; 9 University of West Nusa Tenggara (UNTB), Mataram, Indonesia; Centre for Cellular and Molecular Biology, INDIA

## Abstract

**Background:**

Orangutans are critically endangered primarily due to loss and fragmentation of their natural habitat. This could bring them into closer contact with humans and increase the risk of zoonotic pathogen transmission.

**Aims:**

To describe the prevalence and diversity of *Cryptosporidium* spp., microsporidia and *Giardia intestinalis* in orangutans at seven sites on Sumatra and Kalimantan, and to evaluate the impact of orangutans’ habituation and location on the occurrence of these zoonotic protists.

**Result:**

The overall prevalence of parasites in 298 examined animals was 11.1%. The most prevalent microsporidia was *Encephalitozoon cuniculi* genotype II, found in 21 animals (7.0%). *Enterocytozoon bieneusi* genotype D (n = 5) and novel genotype Pongo 2 were detected only in six individuals (2.0%). To the best of our knowledge, this is the first report of these parasites in orangutans. Eight animals were positive for *Cryptosporidium* spp. (2.7%), including *C*. *parvum* (n = 2) and *C*. *muris* (n = 6). *Giardia intestinalis* assemblage B, subtype MB6, was identified in a single individual. While no significant differences between the different human contact level groups (*p* = 0.479–0.670) or between the different islands (*p* = 0.992) were reported in case of *E*. *bieneusi* or *E*. *cuniculi*, *Cryptosporidium* spp. was significantly less frequently detected in wild individuals (*p* < 2×10^−16^) and was significantly more prevalent in orangutans on Kalimantan than on Sumatra (*p* < 2×10^−16^).

**Conclusion:**

Our results revealed that wild orangutans are significantly less frequently infected by *Cryptosporidium* spp. than captive and semi-wild animals. In addition, this parasite was more frequently detected at localities on Kalimantan. In contrast, we did not detect any significant difference in the prevalence of microsporidia between the studied groups of animals. The sources and transmission modes of infections were not determined, as this would require repeated sampling of individuals, examination of water sources, and sampling of humans and animals sharing the habitat with orangutans.

## Introduction

The orangutan (*Pongo* spp.) is presently classified as endangered. More specifically, the Sumatran orangutan (*Pongo abelii* Lesson, 1827) is red-listed by IUCN, as a critically endangered species (6,600 individuals remaining), and the Bornean orangutan (*Pongo pygmaeus* Linnaeus, 1760) is classified as endangered (54,000 individuals remaining). In the past, orangutans inhabited most of Southeast Asia, but their recent distribution is restricted to the rainforest of northern Sumatra, mainly in Aceh province, and various parts in Kalimantan, including the Indonesian part and portion of Malaysia [1,2]. Orangutan numbers in the wild decreased dramatically over the last century and the population decline has never been more rapid as it is at present [3,4].

Microsporidia, *Giardia* spp. and *Cryptosporidium* spp. are parasitic protists with environmentally resistant stages that can infect a broad spectrum of hosts, including human and non-human primates [5,6]. *Encephalitozoon* spp. and *Enterocytozoon bieneusi*, dangerous pathogens of immunodeficient humans, were previously detected in great apes of the genera *Gorilla* and *Pan* [7–11]. Likewise, *Cryptosporidium* spp. was identified in African great apes in several studies [9,10, 12–14]. Although *Giardia intestinalis* was previously detected in both Bornean and Sumatran orangutans [15,16], there remains a lack of detailed studies on microsporidia and *Cryptosporidium* spp. in captive and wild orangutans.

The main threat to orangutan populations is habitat encroachment by anthropogenic activities, such as logging, conversion of forested land to plantations, forest fires and road building [2,17]. Loss and fragmentation of forests are particularly serious, because forests are the primary habitat for orangutans [2,3]. Opportunistic parasites can pose an important threat to orangutans, which are susceptible to many human pathogens [18–20]. With more human pressure and anthropogenic disturbance in recent years, orangutans are particularly vulnerable. Although many studies have addressed orangutan behavior, ecology and conservation, studies on orangutan susceptibility to parasites are lacking. A major reason for this has been an absence of standard methods for coprology and molecular analyses.

Since there is no study exploring the diversity of gastrointestinal parasitic protists in orangutans using molecular tools, we conducted a comprehensive screening for *Encephalitozoon* spp., *Enterocytozoon bieneusi*, *Cryptosporidium* spp. and *Giardia intestinalis* in several groups of captive, wild and semi-wild orangutans, *Pongo abelii* and *Pongo pygmaeus*, on Sumatra and Kalimantan, Indonesia.

## Materials and Methods

### Ethics Statement

The research complied with the legal requirements for research of Indonesia. A research permit was issued by RISTEK Kementarian Riset dan Teknologi. Permission to collect fecal samples was obtained from LIPI—Lembaga Ilmu Pengetahuan Indonesia (Indonesian Institute of Sciences) and KKH—Kementerian Kehutanan Direktorat Jenderal Perlindungan Hutan dan Konservasi Alam.

Since the collection of fecal samples from orangutans was non-invasive and did not involve interaction with or distress to the animals, the study was not reviewed by an animal ethics committee.

### Study site

**Fig 1:**
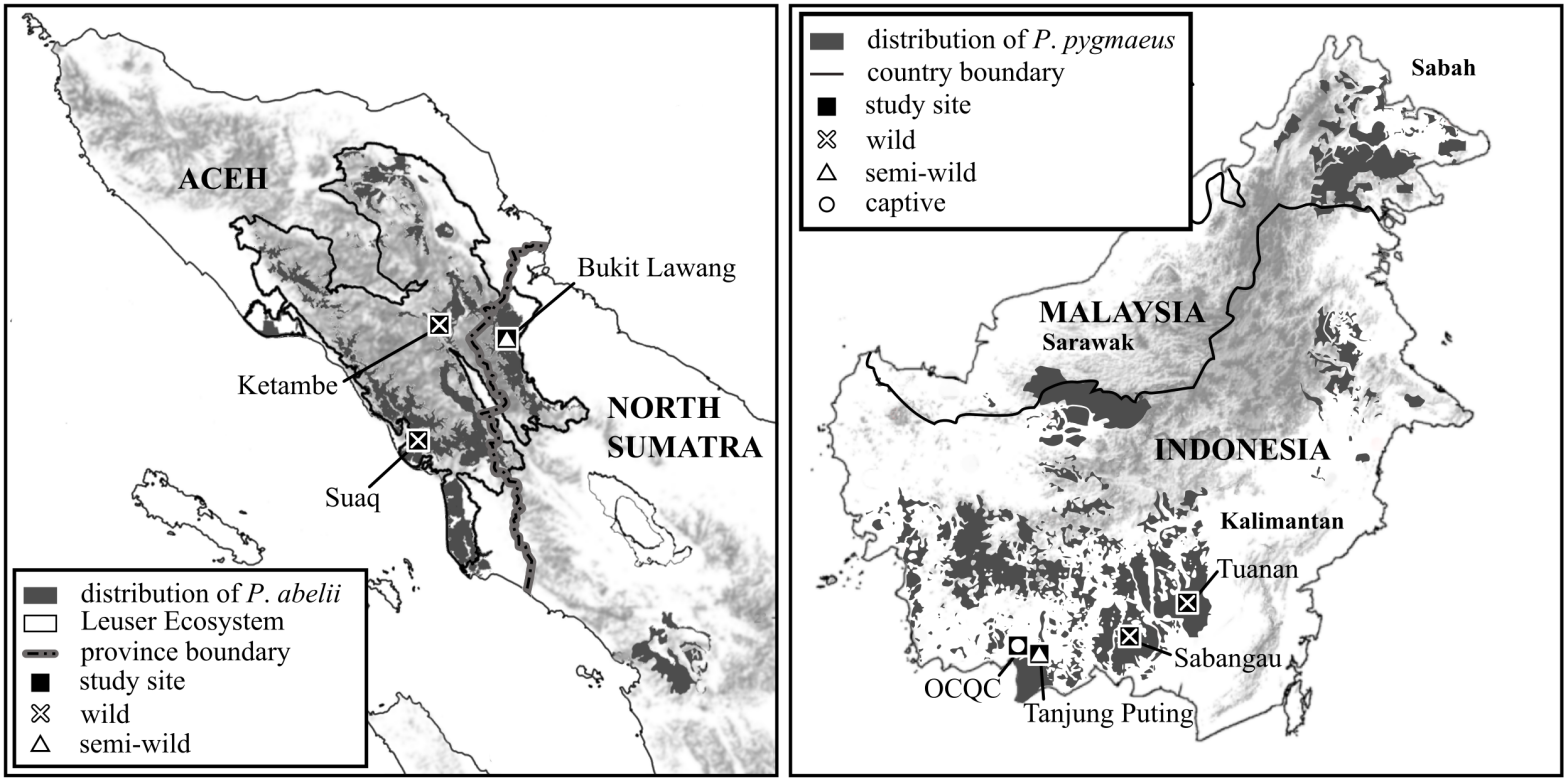
Geographical distribution of *Pongo abelii* and *Pongo pygmaeus* on Sumatra and Kalimantan, respectively, with sampling areas highlighted.

The study was conducted in three localities in Gunung Leuser National Park, Leuser Ecosystem (Sumatra, Indonesia) and four localities in Borneo (South Kalimantan, Indonesia) (Fig 1). These sites are geographically isolated and differ significantly in natural conditions, orangutan habituation and the densities and degrees of human encroachment.

#### Sumatra (*Pongo abelii*)

Bukit Lawang (former Bohorok Orangutan Rehabilitation Centre) is situated in Northern Sumatra, on the eastern border of Gunung Leuser National Park, in hill dipterocarp forest, at an altitude of 500 m above sea level [21]. The temperature in this area ranges between 21 and 28°C. A detailed description of the study area was provided elsewhere [21]. Bukit Lawang hosted a rehabilitation project from 1972 to 2001; therefore, the majority of orangutans (*Pongo abelii*) in this area are semi-wild, released after a reintroduction process. The density of orangutans around Bohorok was 1.8 individuals/km^2^ [4].

Ketambe is situated in Gunung Leuser National Park, Aceh Tenggara, at an altitude of 300–1,000 m above sea level, with a constant temperature of around 28°C. Although the area consists mainly of undisturbed primary rain forest, nearly one fifth was subjected to selective logging from 1999 to 2002 [17]. The orangutan population at this site has been studied since 1971 and was described in other studies [22]. The density of orangutans at this site was 5 individuals/km^2^ [4].

Suaq Balimbing is a coastal swamp situated on the western border of Gunung Leuser National Park, located 65 km from Ketambe at an altitude of 40 m above sea level. and the temperature in this area ranges between 21 and 31°C. More detail describtion was provided elsewhere [23]. The orangutans were found at higher densities (7 individuals/km^2^) and were more likely to form travel parties [24,25], compared to other sample sites.

#### Borneo (*Pongo pygmaeus wurmbii*)

Tuanan is situated in Mawas reserve, Central Kalimantan, close to the Kapuas River, at an altitude of 20–40 m above sea level [4]. The average temperature at this site ranges between 23 and 29°C. The detailed description of this area was listed elsewhere [23]. This area consists of swamp forest and was subjected to selective logging in the early 1990s. The density of orangutans was 4.5 individuals/km^2^ [4].

Sabangau is located in Central Kalimantan and consists of peat swamp forest at an altitude of 10 m above sea level [4]. The mean daily temperature varies from 25 to 33°C [26]. A more detailed description of the study area was provided [26]. The site was selectively logged form 1966 to 1996, followed by illegal logging from 1996 to 2004 [27]. The density of orangutan was 2.3 individuals/km^2^ [4].

Orangutan Care and Quarantine Center (OCQC) is situated close to Tanjung Puting National Park in Central Kalimantan. The Centre housed about 270 orangutans, confiscated by Indonesian authorities [28], in cages.

Tanjung Puting National Park is situated in the province of Kalimantan Tengah at an altitude of 10 m above sea level [4]. The temperature varies from 22 to 32°C. The study area was described in more detail [29]. The first Kalimantan orangutan rehabilitation program was established at Camp Leaky in Tanjung Putting. Although the rehabilitation process no longer takes place at this location, rehabilitated individuals and the second generation of their offspring can be found in the area surrounding Camp Leaky [30]. The density of orangutans was 2.7 individuals/km^2^ [4].

### Orangutan groups studied and sample collection

Individuals from all localities are habituated, thus accustomed to human presence, and ethology data are collected. For the purpose of this study were animals grouped based on the degree of human contact and designated i) wild, representing orangutans that have no physical contact with humans, and live in nature without human control and care (Sabangau, Tuanan, Suaq, Ketambe); ii) semi-wild, released after a reintroduction process, and the density of humans was higher than at other sites, so there was increased possibility of contact between humans and orangutans (Bukit Lawang, Tanjung Putting National Park); and iii) captive, having the highest human contact since orangutans are kept in cages (Orangutan Care and Quarantine Center).

Fecal samples from orangutans were collected yearly from 2002 to 2011. All samples were obtained immediately after defecation from identified individuals as part of a long-term orangutan heath monitoring project. Some individuals were sampled repeatedly (maximum of five times). Each sample was preserved in 96% ethanol in labeled and Parafilm-sealed examination tubes, and transported to the laboratory.

### DNA extraction, PCR amplification, sequencing and genotyping

The suspension of each fecal sample in alcohol was evaporated overnight at 60°C. A total of 200 mg of feces was homogenized by bead disruption using 0.5 mm glass beads (Biospec Products, Inc., Bartlesville, OK, USA) in a FastPrep^®^-24 Instrument (MP Biomedicals, Santa Ana, CA, USA) at a speed of 5 m/s for 1 min, followed by isolation/purification using the QIAamp^®^ DNA Stool Mini Kit in accordance with the manufacturer’s instructions (Qiagen, Hilden, Germany). Purified DNA was stored at -20°C prior to use in polymerase chain reaction (PCR). All DNA samples obtained for the study were analyzed by PCR using sets of genus-specific primers. A nested PCR approach was used to amplify a region of the internal transcribed spacer (ITS) of *Enterocytozoon bieneusi* (~390 bp) [31], the small ribosomal subunit rRNA gene (SSU) of *Cryptosporidium* spp. (~ 830 bp) [32], and the triosephosphate isomerase gene (TPI) of *Giardia intestinalis* (~500 bp) [33]. The following primers sets were used to amplify *Encephalitozoon* spp.: the int580f and int580r primer set for primary PCR analysis [34] and the MSP3 and MSP4 primer set for secondary PCR (~320 bp) [35]. All secondary PCR products were run on a 2% agarose gel containing 0.2 μg/ml ethidium bromide in 1×TAE buffer. PCR products of the predicted size were visualized using a UV light source, cut from the gel, and then extracted using QIAquick Gel Extraction Kit (Qiagen, Hilden, Germany). Gel purified secondary products were sequenced in both directions with an ABI 3130 genetic analyzer using the secondary PCR primers and the BigDye1 Terminator V3.1 cycle sequencing kit (both Applied Biosystems, Foster City, CA, USA) in 20 μl reactions.

Positive and negative (PCR water) controls were included in each analysis. DNA from *E*. *intestinalis* spores, grown *in vitro* in the Laboratory of Veterinary and Medical Protistology at Institute of Parasitology, Biology Centre, Czech Academy of Sciences, from *E*. *bieneusi* spores of genotype S6, originally isolated from a house mouse, from *Cryptosporidium serpentis*, originated from a corn snake, and from *Giardia intestinalis* assemblage E, originated from a domestic goat, were used as positive controls for appropriate PCR reactions. All samples were analyzed in duplicate. In case of positive detection, DNA was re-isolated from the sample and the finding was independently verified.

### Phylogenetic analyses

The nucleotide sequences of each gene obtained in this study were assembled and edited using the software ChromasPro 1.7 (Technelysium, Pty, Ltd.) and aligned with previously published sequences in MAFFT version 7 online server with automatic selection of alignment mode (http://mafft.cbrc.jp/alignment/server/).

Alignment adjustments were made manually to remove artificial gaps using BioEdit. Sequences from this study have been deposited in GenBank under the accession numbers KP994659-KP994665. Phylogenetic analyses of aligned sequences was inferred using the maximum likelihood method [36], with the substitution model that best fit the alignment selected using the Bayesian information criterion in the program MEGA6 [37]. A bootstrap consensus tree was inferred from 1000 pseudoreplicates. Phylogenetic trees were drawn using the MEGA6 and edited for style using CorelDrawX7 Graphics Suite (Ottawa, Ontario, Canada).

### Statistical analyses

All computation was carried out with the programming environment R 3.0.2. More specifically, generalized linear mixed model (GLMM) with binominal distribution was fitted to avoid bias from repeated sampling in a significant number of animals. Using this model, we analyzed the relationship between the occurrence of parasites (*Cryptosporidium* spp., *Encephalitozoon* spp. or *Enterocytozoon bieneusi*) and the independent variables, including island (Kalimantan, Sumatra) and level of human-ape contact (captive, semi-wild, wild). For the analyses, the random factors “location” and “individual” were nested into the fixed factors “level of human-ape contact” and “island”. Due to a low number of cases, similar analyses for *Giardia* were not performed.

## Results

Out of 298 examined animals, 33 were positive for the tested parasites at least once during the study (11.1%). The most frequently detected parasite was *Encephalitozoon cuniculi* genotype II, which was identified in 21 animals (7%), followed by *Cryptosporidium* spp., identified in eight animals (2.6%), *Enterocytozoon bieneusi*, identified in six animals (2.0%), and *Giardia* spp., identified in a single animal (0.3%) (Table 1). Only *E*. *cuniculi* was detected in the same individuals on multiple occasions (S1 Table).

**Table 1:** *Encephalitozoon cuniculi*, *Cryptosporidium* spp., *Enterocytozoon bieneusi* and *Giardia* spp. in orangutans under different levels of human contact.

	Locality	Human contact	n1	n2	Positive animals			
					*Encephalitozoon* spp.	*Enterocytozoon bieneusi*	*Cryptosporidium* spp.	*Giardia* spp.
**Kalimantan**	Sabangau	wild	18	39	-	-	-	-
	Tuanan	wild	15	48	1× EC II^#^	1× D^#^	-	-
	OCQC	captive	135	141	-	1×Pongo 2^$^	5× *C*. *muris*^$^ 1× *C*. *parvum* type A	-
	Tanjung Putting	semi-wild	40	40	1× EC II	1× D	1× *C*. *parvum* type B	
**Sumatra**	Bukit Lawang	semi-wild	56	114	10× EC II	1× D	-	1× B
	Suaq	wild	17	26	4× EC II	-	1× *C*. *muris*	-
	Ketambe	wild	17	62	5× EC II^&^	2× D^&^	-	-
			**298**	**470**	**21**	**6**	**8**	**1**

D = *E*. *bieneusi* genotype D; EC II = *Encephalitozoon cuniculi* genotype II; B = *Giardia intestinalis* assemblage B; n1 = number of sampled animals; n2 = number of samples;

^#, &, $^co-infection in one animal

**Fig 2:**
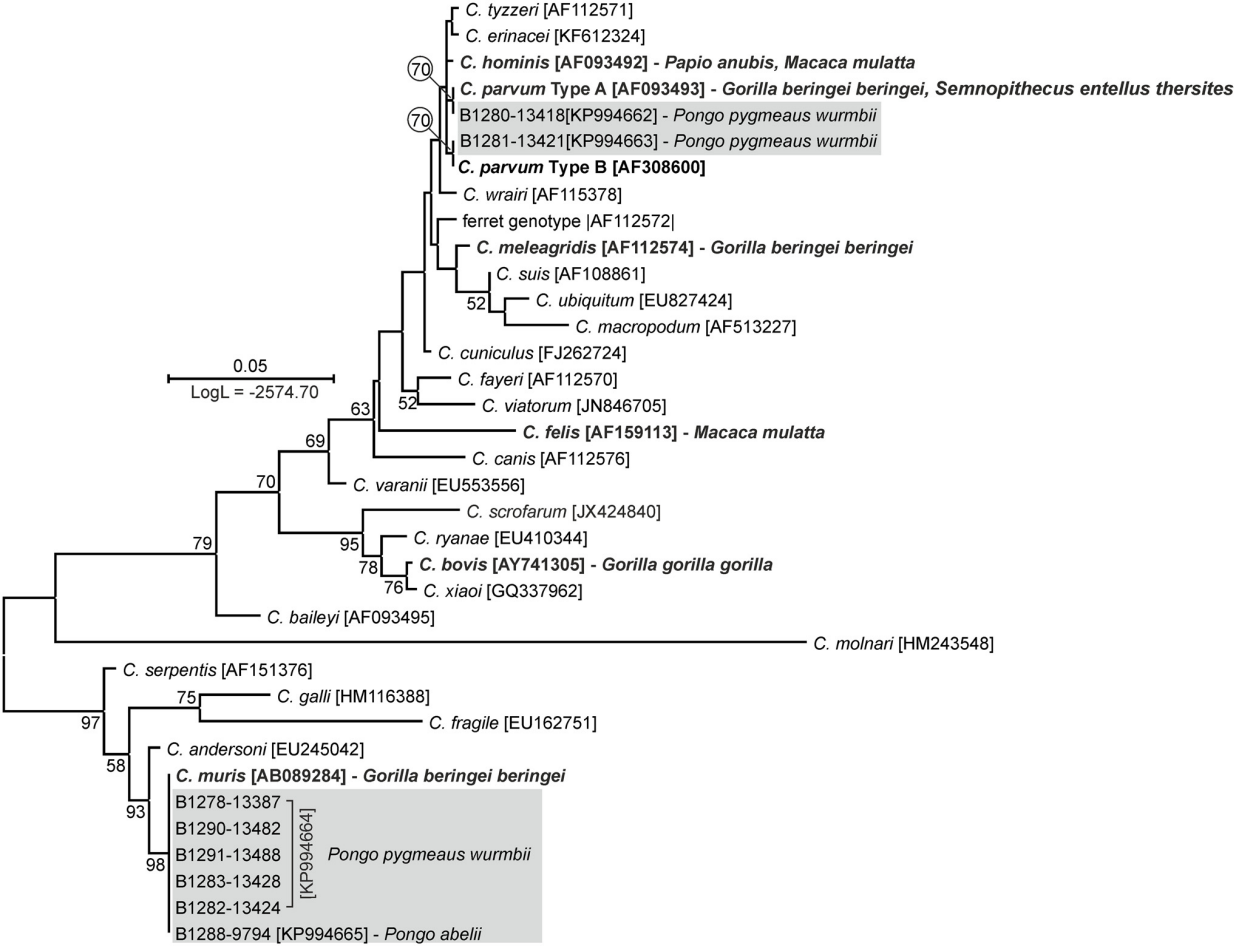
Maximum-likelihood tree of partial sequences of small ribosomal subunit (SSU) gene of *Cryptosporidium* spp. Sequences generated in this study are shaded. Taxa previously found in non-human primates are bolded. The tree with the highest log likelihood is shown. The percentage of trees in which the associated taxa clustered together is shown next to the branches. Only bootstrap values >50% are shown. Scale bar included in each tree.

*Cryptosporidium* SSU sequences from six animals were 100% identical to a *C*. *muris* nucleotide sequence originally isolated from a rat (AB089284). Sequences from two animals clustered with *C*. *parvum* in a ML phylogeny. One of the sequences was identical to the predominant Type A SSU paralog, while the other was identical to the Type B paralog, which is less frequently reported in the literature [38] (Fig 2).

**Fig 3:**
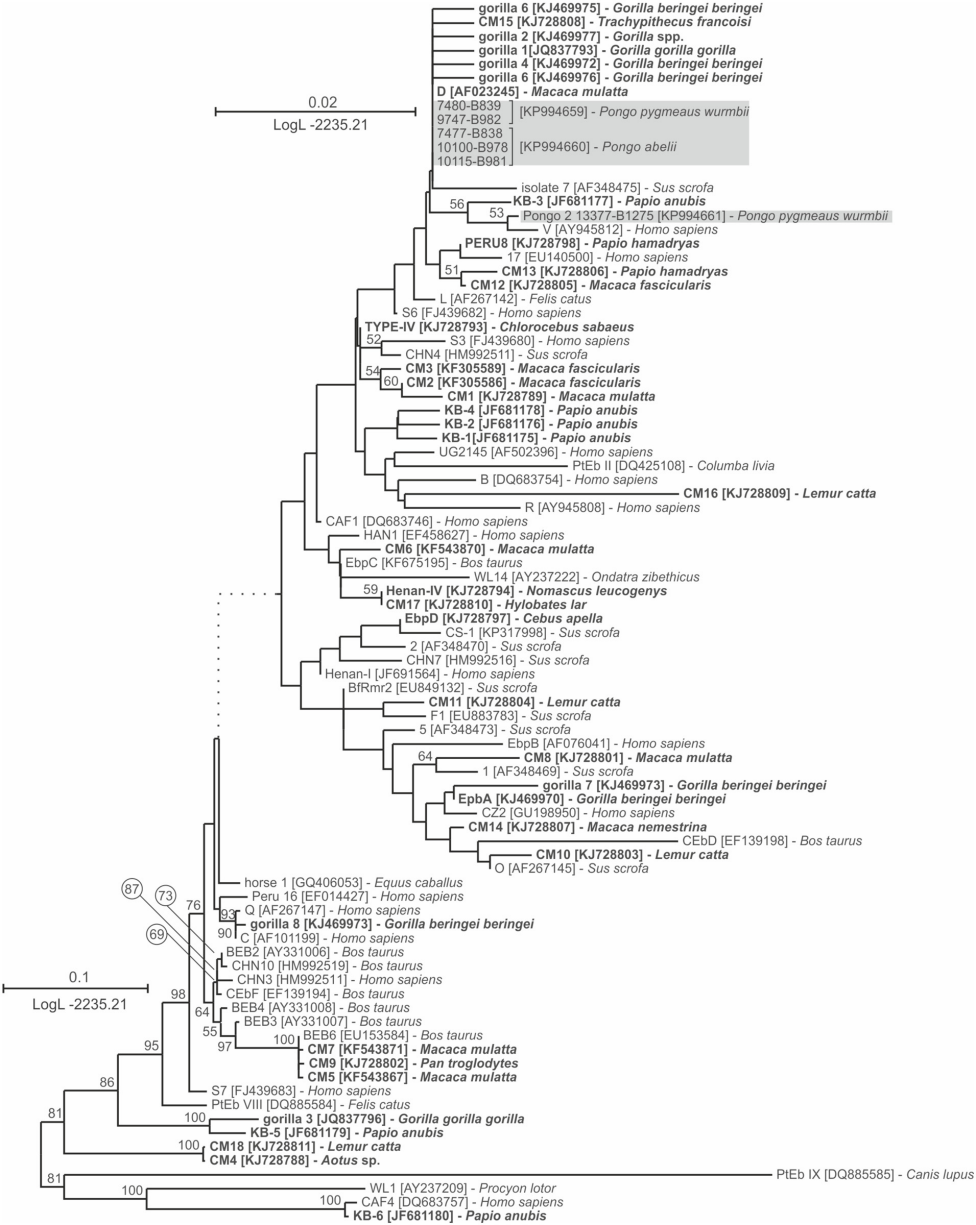
Maximum-likelihood tree of whole ITS region of *Enterocytozoon bieneusi*. Sequences generated in this study are shaded. Taxa previously found in non-human primates are bolded. The tree with the highest log likelihood is shown. The percentage of trees in which the associated taxa clustered together is shown next to the branches. Only bootstrap values >50% are shown. Scale bar included in each tree.

Analyses of the alignments of microsporidial ITS sequences showed 21 isolates with 100% nucleotide similarity to *E*. *cuniculi* genotype II (GQ422153, data not shown). Five isolates positive for *E*. *bieneusi* were identified as genotype D (identical to AF101200). In addition, one sequence was identified as novel genotype Pongo 2 (KP994661). This genotype is closely related to *E*. *bieneusi* genotype D, sharing 99.1% nucleotide similarity. The global topology of the *E*. *bieneusi* genotype tree is shown in Fig 3.

The *Giardia* TPI gene was amplified from a single sample and the sequence (KR011753) shared 99.8% nucleotide similarity with *Giardia intestinalis* assemblage B, subtype MB6 (KF679740) (phylogenetic analyses not shown).

Although a single-species infection was detected in most examined animals, two cases of co-infection with *E*. *cuniculi* genotype II and *E*. *bieneusi* genotype D were detected. Moreover, co-infection with *E*. *bieneusi* genotype Pongo 2 and *Cryptosporidium muris* was identified in one case (Table 1). Parasites were observed in each studied localities with the exception of Sabangau.

There was no significant difference in *Cryptosporidium* spp. prevalence between captive and semi-wild individuals (*p* = 0.838, z = -0.2). However, compared to other groups, wild individuals were significantly less frequently infected by *Cryptosporidium* spp. (*p* < 2×10^−16^, z = -226.05). Regarding the occurrence of this parasite on two different islands, *Cryptosporidium* spp. was significantly more prevalent in orangutans on Kalimantan than on Sumatra (*p* < 2×10^−16^, z = -110.2).

The prevalence of *E*. *bieneusi* or *E*. *cuniculi* did not differ significantly between the different human contact level groups (captive, semi-wild or wild) (*p* = 0.479–0.670, z = 0.426–0.707) or between the different islands (*p* = 0.992, z = 0.010).

## Discussion

Great apes and humans, due to their high genomic similarity and close evolutionary relationship, share a wide range of common pathogens [39,40]. Therefore, apes could be an important source of emerging human pathogens [40–43], and humans could be a source of pathogens in apes [18,42]. Currently, orangutans (*Pongo* spp.) and gorillas *(Gorilla gorilla*) are the most endangered great apes, primarily due to anthropogenic forest disturbance, trade and hunting. Consequently, several conservation strategies have been implemented. Rehabilitation and reintroduction of confiscated animals and protection of their forest habitats are critical orangutan conservation strategies that could help to replenish populations [44]. However, rehabilitated individuals, which are often kept under unnatural conditions that are against their semi-solitary nature, may be more susceptible to pathogen transmission [16]. Moreover, introduction of human pathogens to the naïve orangutan population could pose a serious risk to these endangered animals [18,42].

To the best of our knowledge, this is the first report of *Encephalitozoon* spp. and *Enterocytozoon bieneusi* in orangutans, although there have been several reports of these parasites in African great apes [7–11]. There have been studies on microsporidia in Malaysia and Indonesia, but these have been limited to human populations, primarily aboriginal, and have not identified sources of infection or modes of transmission [45–50].

In our study, the prevalence of *E*. *cuniculi* reached 7%, which is the same as previously reported in western lowland gorillas, and slightly lower than the 11% prevalence reported in mountain gorillas living in areas of high human densities [9, 10]. Surprisingly, we did not detect *E*. *cuniculi* genotype I, described as the predominant genotype in chimpanzees and gorillas [9,10]. We also failed to detect two other species of the genus *Encephalitozoon*, *E*. *intestinalis* and *E*. *hellem*, which were described in African apes [7,8,11].

The observed mean prevalence of *Enterocytozoon bieneusi* was 2% (0–11.7%) which is similar to the 2.7% prevalence reported in captive chimpanzees, and is lower than 18% prevalence reported in mountain gorillas [8,9]. In this study, *E*. *bieneusi* genotype D and novel genotype Pongo 2 was detected. The novel genotype Pongo 2 clustered with human specific genotype V [51] and with most genotypes reported from African apes, including gorilla 1–2 and gorilla 4–8, EpbA and C genotype, which belong to group 1 based on standardized nomenclature [8,9,52]. Group 1 includes most of the previously reported *E*. *bieneusi* isolates from humans, including zoonotic isolates; therefore, the host specificity of genotypes detected in apes is unclear. We found that the prevalence of microsporidia did not differ among wild, semi-wild and captive animals, which is consistent with previous studies on African great apes [9,10].

To our knowledge, this is the first study to describe the molecular characteristics of *Cryptosporidium* spp. infecting orangutans. *Cryptosporidium* spp. was detected in eight individuals (2.7%) with most of these cases occurring at the Orangutan Care and Quarantine Center. There have been reports on *Cryptosporidium* spp. in Asian apes [20,53], but parasites were identified by oocyst morphology without further molecular characterization. Based on a number of studies, *Cryptosporidium* spp. oocysts are ubiquitous in Southeast Asia, occurring in water, livestock, wildlife and rural and urban human populations [54–57]. However, most of these studies were focused on cryptosporidiosis of HIV positive human, as with the raising numbers of HIV cases in Malaysia and Indonesia, the significance of the studies of opportunistic protists are continually increasing [50,58–61].

Although there have been many reports of *Cryptosporidium* spp. in non-human primates [11,14,53,62–74], few have provided detailed molecular information about these parasites in the great apes [9–12,75]. Paralogous copies of the *C*. *parvum* SSU gene, Type A and Type B, were detected in the present study. Type A is present at a higher copy number than Type B in the *C*. *parvum* genome [38] and is therefore more frequently detected; however, the Type B paralog also has been reported in humans, both immunocompetent and HIV-positive, capybara (*Hydrochoerus hydrochaeris*), leopard gecko (*Eublepharis macularius*) and turkeys [76–80]. Similar to previous studies on great apes, *C*. *parvum* infection was detected only in animals with closer contact with human, e.g. semi-wild and captive [75]. Host specific *Cryptosporidium* species, including *C*. *bovis*, *C*. *meleagridis*, and *C*. *muris*, have been detected rarely in gorillas [9,10]. Similar to [9], where *C*. *muris* in research groups of mountain gorillas, which were in increased contact with human was reported, five of the six *C*. *muris* isolates in this study were detected in animals kept in the Orangutan Care and Quarantine Center. Although natural *C*. *muris* infections have been reported in human and non-human primates [67,81], discrimination of mechanical passage of ingested oocysts from ongoing infection is almost impossible. The higher prevalence of *C*. *muris* in captive individuals could be due to increased contact with fecal samples from rodents, natural hosts of *C*. *muris* [82], at the care centre. Our findings are in agreement with those of previous studies, and they suggest that non-human primates with high levels of human contact are more frequently infected with *Cryptosporidium* [53, 64–67].*Cryptosporidium* sp. was significantly more prevalent in orangutans on Kalimantan than Sumatra. This could be due to greater levels of contact with humans, wildlife and domestic animals experienced by Kalimantan orangutans.

To date, at least eight assemblages of *G*. *intestinalis* have been described and these vary significantly in degree of host specificity [5]. Assemblage A and B are zoonotic and can infect a broad range of hosts, including humans, non-human primates, domestic animals and wildlife. The other assemblages (C to H) are considered to be highly host-specific [5,83]. However, assemblage E, which is specific for artiodactyls, also has been reported in non-human primates [84]. Previous studies on *Giardia* in orangutans did not include the molecular characterization necessary for assemblage distinction [15,16]. *Giardia* sp. has been described in slow loris (*Nycticebus* spp.), rhesus macaque and rats (*Rattus* spp.), animals whose habitat overlaps with orangutans [67,85]. In our study, a single case (0.36%) of *G*. *intestinalis* was identified in Bukit Lawang, a former rehabilitation centre, and an area where orangutans have contact with humans. This rare occurrence is consistent with the very low or zero prevalence described previously in the wild African great apes [9,10,13]. The TPI gene was chosen because *Giardia intestinalis* exhibits high genetic variability at this locus [86]. Phylogenetic analyses based on TPI gene sequences showed that our isolate belonged to assemblage B subtype MB6, which was recently described in a captive rhesus macaque (*Macaca mulatta*) at a monkey farm in China [67]. This is consistent with the predominance of assemblage B in human and non-human primates, although assemblage A has been reported in African great apes [10,13,83]. A number of studies have speculated about the occurrence of human-to-animal transmission of *G*. *intestinalis*, but this remains to be confirmed [84,87,88]. Nevertheless, these pathogens are known to reach a high prevalence in the human populations of some rural regions of Malaysia and Indonesia, which could increase the risk of transmission to animals living in close contact with humans [89–91].

Our results showed the presence of potentially zoonotic protists at almost all studied sites. Microsporidia (*Encephalitozoon* spp. and *Enterocytozoon bieneusi*) were detected in orangutans for the first time and molecular data were obtained on *Cryptosporidium* spp. and *G*. *intestinalis* in this host. However, questions remain about the sources and transmission modes of these parasites in Asian great apes, and these must be answered with further studies on wildlife, livestock, humans, and water in environments inhabited by orangutans. Also, the effect of these protists on orangutan health needs to be resolved.

## Supporting Information

S1 Table The list of screened samples from positive animals. (DOC)
