# Supplementary material for: Prevalence of Cryptosporidium spp., Enterocytozoon bieneusi, Encephalitozoon spp. and Giardia intestinalis in Wild, Semi-Wild and Captive Orangutans (Pongo abelii and Pongo pygmaeus) on Sumatra and Borneo, Indonesia
Source: PLoS One. 2016 Mar 31;11(3):e0152771. doi: 10.1371/journal.pone.0152771 (PMC4816420; doi:10.1371/journal.pone.0152771)
Supplement: S1 Table — (DOC) [file pone.0152771.s001.doc]

**S1 Table. The list of screened samples from positive animals.**

| **Locality** | | **Animal identification code** | ***E*. *cuniculi* genotype II** | ***E*. *bieneusi*** | | ***C*. *muris*** | ***C*. *parvum*** | | ***G*. *intestinalis* genotype B** |
| --- | --- | --- | --- | --- | --- | --- | --- | --- | --- |
|  |  |  |  | **genotype D** | **Pongo 2** |  | **type A** | **type B** |  |
| **Kalimantan** | **Tuanan** | TU011 | 7443, 7442, 7482, 9745, 9747 * | 7443, 7442, 7482, 9745, 9747 * |  |  |  |  |  |
|  | **OCQC** | OC005 |  |  | 13377 | 13387 |  |  |  |
|  |  | OC023 |  |  |  |  | 13418 |  |  |
|  |  | OC062 |  |  |  | 13424 |  |  |  |
|  |  | OC072 |  |  |  | 13428 |  |  |  |
|  |  | OC106 |  |  |  | 13482 |  |  |  |
|  |  | OC121 |  |  |  | 13488 |  |  |  |
|  | **Tanjung-Puting** | TP037 | 13508 |  |  |  |  |  |  |
|  |  | TP003 |  | 7480 |  |  |  |  |  |
|  |  | TP015 |  |  |  |  |  | 13421 |  |
| **Sumatra** | **Bukit Lawang** | BL003 | 7407, 9731, 9807, 9948, 10004 |  |  |  |  |  |  |
|  |  | BL008 |  | 7476, 7477, 9925, 9943, 10000 |  |  |  |  |  |
|  |  | BL039 |  |  |  |  |  |  | 9927, 9928, 9929, 9930, 9931 |
|  |  | BL004 | 7408, 9805, 9834, 9910 |  |  |  |  |  |  |
|  |  | BL006 | 7466, 7467 |  |  |  |  |  |  |
|  |  | BL012 | 7478, 9440, 9860, 9939, 10010 |  |  |  |  |  |  |
|  |  | BL023 | 9719, 7420, 7424, 9826, 9850 |  |  |  |  |  |  |
|  |  | BL025 | 9802, 9810, 9824, 9831, 9836 |  |  |  |  |  |  |
|  |  | BL026 | 9804, 9806, 9808, 9811, 9812 |  |  |  |  |  |  |
|  |  | BL031 | 9843 |  |  |  |  |  |  |
|  |  | BL033 | 9845 |  |  |  |  |  |  |
|  |  | BL034 | 9846, 9853 |  |  |  |  |  |  |
|  | **Suaq** | SU003 | 9789 |  |  |  |  |  |  |
|  |  | SU013 |  |  |  | 9794, 9901 |  |  |  |
|  |  | SU005 | 9791 |  |  |  |  |  |  |
|  |  | SU012 | 9793, 9801 |  |  |  |  |  |  |
|  |  | SU015 | 9796, 9781 |  |  |  |  |  |  |
|  | **Ketambe** | KE016 | 10108, 10109, 10111, 10115 * | 10108, 10109, 10111, 10115 * |  |  |  |  |  |
|  |  | KE002 | 7448, 7449, 9861, 9864, 9872 |  |  |  |  |  |  |
|  |  | KE014 |  | 9903, 10088, 10099, 10102, 10100 |  |  |  |  |  |
|  |  | KE006 | 7458, 9711, 9718, 9866, 9876 |  |  |  |  |  |  |
|  |  | KE009 | 9803, 9865, 9869, 9887, 9895 |  |  |  |  |  |  |
|  |  | KE012 | 9877, 9713, 10090 |  |  |  |  |  |  |

*co-infection in one animal; positive samples highlighted in red
